# Schizophrenia Related Variants in *CACNA1C* also Confer Risk of Autism

**DOI:** 10.1371/journal.pone.0133247

**Published:** 2015-07-23

**Authors:** Jun Li, Linnan Zhao, Yang You, Tianlan Lu, Meixiang Jia, Hao Yu, Yanyan Ruan, Weihua Yue, Jing Liu, Lin Lu, Dai Zhang, Lifang Wang

**Affiliations:** 1 Institute of Mental Health, The Sixth Hospital, Peking University, Beijing, China; 2 Key Laboratory of Mental Health, Ministry of Health & National Clinical Research Center for Mental Disorders (Peking University), Beijing, China; 3 Peking-Tsinghua Center for Life Sciences, Peking University, Beijing, P. R. China; 4 PKU-IDG/McGovern Institute for Brain Research, Peking University, Beijing, P. R. China; Albert Einsten College of Medicine, UNITED STATES

## Abstract

Autism spectrum disorder (ASD) is a group of neurodevelopmental disorders with a strong genetic component. Many lines of evidence indicated that ASD shares common genetic variants with other psychiatric disorders (for example, schizophrenia). Previous studies detected that calcium channels are involved in the etiology of many psychiatric disorders including schizophrenia and autism. Significant association between *CACNA1C* (calcium channel, voltage-dependent, L type, alpha 1C subunit) and schizophrenia was detected. Furthermore, rare mutation in *CACNA1C* is suggested to cause Timothy syndrome, a multisystem disorder including autism-associated phenotype. However, there is no evidence for association between *CACNA1C* and autism in Chinese Han population. To investigate the association between single nucleotide polymorphisms (SNP) in *CACNA1C* and autism, we first performed a family-based association study between eighteen SNPs in *CACNA1C* and autism in 239 trios. All SNPs were genotyped by using Sequenom genotyping platform. Two SNPs (rs1006737 and rs4765905) have a trend of association with autism. To further confirm the association between these two SNPs with autism, we expanded the sample size to 553 trios by adding 314 trios. Association analyses for SNPs and haplotype were performed by using family-based association test (FBAT) and Haploview software. Permutation tests were used for multiple testing corrections of the haplotype analyses (n=10,000). The significance level for all statistical tests was two-tailed (*p*<0.05). The results demonstrated that G allele of rs1006737 and G allele of rs4765905 showed a preferential transmission to affected offspring in 553 trios (*p*=0.035). Haplotype analyses showed that two haplotypes constructed from rs1006737 and rs4765905 were significantly associated with autism (*p*=0.030, 0.023, respectively; Global *p*=0.046). These results were still significant after permutation correction (n=10,000, *p*=0.027). Our research suggests that *CACNA1C* might play a role in the genetic etiology of autism in Chinese Han population.

## Introduction

Autism is a neurodevelopmental disorder characterized by deficits in social interaction and communication, and the presence of repetitive or stereotypic behaviors [1]. These symptoms become apparent in the first three years of life. Twin studies have provided evidence for a strong genetic component for autism. The concordance rate for monozygotic twins is much higher than that for dizygotic twins (70%-82% vs. 0%-10%). The estimated heritability of autism is more than 90% [2]. The contribution of common variants is not only substantial but also highly polygenic. By analyzing common variations throughout the genome, a recent study showed that common variations, individually of small effect, exert substantial additive genetic effects on autism spectrum disorder (ASD) liability [3]. It provides evidence for the common disease-common variant hypothesis.

Calcium channels mediate the influx of calcium ions into the cell upon membrane polarization. *CACNA1C* (calcium channel, voltage-dependent, L type, alpha 1C subunit) encodes an alpha-1 subunit of a voltage-dependent calcium channel, which plays an important role in the development and function of the central nervous system. CACNA1C influences neuronal firing by modulating calcium channel functions. Moreover, it modulates γ-aminobutyric acid-transmitting interneuron function. Therefore, CACNA1C could affect brain regional activation and inter-regional connectivity [4]. Mice with a selective inactivation of C*acna1c* gene in the hippocampus and neocortex show a defect in N-methyl-D-aspartate (NMDA) receptor-independent long-term potentiation in the CA1 region of the hippocampus that paralleled by a severe memory deficit. It indicated that CACNA1C may play a role in NMDA receptor-independent synaptic plasticity in hippocampus [5].

A previous meta-analysis of genome-wide association study (GWAS) identified that single nucleotide polymorphism (SNP) rs1006737 in *CACNA1C* was significantly associated with bipolar disorder (*p* = 7.0×10^−8^) [6]. While within the Wellcome Trust Case Control Consortium (WTCCC) bipolar disorder dataset, the significant level was *p* = 7.0×10^−4^ [7]. Another GWAS showed that rs1006737 was associated with bipolar disorder (OR = 1.21) [8]. Furthermore, *CACNA1C* was also associated with schizophrenia. One study found that the risk allele of rs1006737 conferred increasing risk for schizophrenia (*p* = 0.034) [9]. A previous GWAS demonstrated that rs4765905 in *CACNA1C* reached genome-wide significance in 16,374 cases with schizophrenia, schizoaffective disorder or bipolar disorder and 14,044 controls (*p* = 7.0×10^−9^) [10]. Recently, another independent GWAS identified that rs4765905 in *CACNA1C* was associated with schizophrenia (*p* = 1.23×10^−8^) [11]. Moreover, other independent studies replicated the association of the specific SNP rs1006737 in *CACNA1C* with schizophrenia in white subjects of self-identified European descent, Danish subjects, and Spanish population, respectively [12–14]. All these studies provide genetic evidence that *CACNA1C* may play a role in the etiology of psychiatric disorders.

For clinical features, autism and schizophrenia share the same neurocognitive defects such as impaired executive function and deficits in social functioning [15,16]. Some of the SNPs conferring risk for schizophrenia also appear to confer risk for autism. There is genetic evidence of shared loci and pathways in the genetic etiology of autism and schizophrenia [11,17–21]. A recent study published by the Psychiatric Genomics Consortium (PGC) has identified that several SNPs (including SNPs in *CACNA1C*) were significantly associated with five major psychiatric disorders including ASD, attention deficit-hyperactivity disorder, bipolar disorder, major depressive disorder, and schizophrenia in 33,332 cases and 27,888 controls of European ancestry [22]. These findings provide further support for some degree of overlap in the susceptibility to mental illness across schizophrenia and autism.

Genetic support of a role for calcium channel genes in ASD is the association of two SNPs (rs757415 and rs12603112) in *CACNA1G* encoding a T-type Ca^2+^ channel subunit [23]. Rare mutation in *CACNA1C* is suggested to cause Timothy syndrome, a disorder whose features include ASD-related phenotypes and intellectual disability [24]. A previous GWAS suggest that SNPs surrounding *CACNA1C* show suggestive evidence of association with ASD [25]. These studies indicate that calcium channels might be involved in the etiology of autism. However, no replication association studies have yet been reported.

To investigate whether the genetic variants in *CACNA1C* are associated with autism, we performed a family based association study between *CACNA1C* and autism in Chinese Han population. Here we report the association of two SNPs (rs1006737 and rs4765905) and haplotypes in *CACNA1C* with autism. These results suggest that *CACNA1C* may be a susceptibility gene of autism.

## Materials and Methods

### Ethics statement

This research was approved by the Ethics Committee of Institute of Mental Health, The Sixth Hospital, Peking University. All subjects provided written informed consents, and informed written consents for children were obtained from their biological parents (the children’s legal guardians).

### Subjects

Our study included 553 children affected with autism and their biological parents of Chinese Han descent. These trios were recruited at the Institute of Mental Health, Peking University, China. In the first discovery sample, we recruited 239 autism trios. Among the children affected with autism, 226 were male and 13 were female. The age of these children at the clinical assessment time ranged from 2 to 17 years old and the mean age was 7.5 years old. Then, we expanded our sample to 553 trios (1659 individuals) by recruiting additional 314 trios (median age of autistic children was 6.0 years old). Among all 553 autistic children, 513 were male and 40 were female. The assessments of autism were established by two senior psychiatrists using DSM-IV criteria. Autism Behavior Checklist (ABC) [26] and Childhood Autism Rating Scale (CARS) [27] were used for additional clinical assessment. All children had scored more than 53 for ABC and 35 for CARS scales. Exclusion criteria included children with phenylketonuria, fragile X syndrome, tuberous sclerosis, chromosomal abnormality by karyotyping analysis, and non-Han Chinese ancestry. To decrease the heterogeneity, children affected with Asperger disorder and Rett syndrome were excluded in our study.

Blood was obtained from autistic children and their biological parents after informed contents were obtained.

### SNP selection and genotyping

Eighteen SNPs with minor allele frequency (MAF) >0.05 in *CACNA1C* were selected. These SNPs were distributed from 2011392bp to 2668602bp on chromosome 12 (cover 90.4% of the *CACNA1C* region) with a mean inter-SNP distance of 38.7 Kb (GRCh38, National Center for Biotechnology Information [NCBI]). Among these 18 SNPs, rs1006737 and rs476590 were selected for positive association with schizophrenia and bipolar in previous studies. Furthermore, Genotype data in Chinese Han in Beijing (CHB) from the HapMap phase II and III was downloaded from Hapmap genotype dataset (http://hapmap.ncbi.nlm.nih.gov/). Then pair-wise tagging in the Tagger module in Haploview version 4.2 program (http://www.broad.mit.edu/mpg/haploview/) was considered to select these SNPs that could capture the known common genetic variation.

Genomic DNA was extracted from blood using Qiagen QIAamp DNA Kits. All SNPs were genotyped using Sequenom genotyping platform, which uses the MALDI-TOF primer extension assay. Primers were designed according to the sequence of the forward strand from dbSNP database (http://www.ncbi.nlm.nih.gov/SNP/). We used iPlex genotyping assay, which has increased plexing efficiency and flexibility for the MassARRAY system through single base primer extension with mass-modified terminators [28–30].

To confirm the genotype results by Sequenom genotyping platform, all these eighteen SNPs were re-genotyped in 10% of the whole samples.

### Statistical analysis

To decrease population stratification, we performed a family based association study. All those SNPs with MAF greater than 5% were used as genetic markers in this study. The Hardy-Weinberg Equilibrium (HWE) for genotype frequency distributions was tested by using the chi-square goodness-of-fit test. Mendelian inconsistencies were checked using family-based association test (FBAT) software v1.7.2 (http://www.biostat.harvard.edu/~fbat/default.html) [31]. Genotypes of families with Mendelian errors have been reset to zero.

Association analyses for SNPs and haplotype were performed by using FBAT software. Single marker association tests were performed under an additive model. The FBAT program uses generalized score statistics to perform a variety of transmission disequilibrium tests (TDT), including haplotype analyses. Moreover, the global haplotype tests of association were performed under “multiallelic” mode in haplotype based association test (HBAT). Meanwhile, the individual haplotype tests were conducted under “biallelic” mode in HBAT. Permutation tests were used for multiple testing corrections of the haplotype analyses (n = 10,000). The significance level for all statistical tests was two-tailed (*p*<0.05). Haploview software provides estimation of pairwise linkage disequilibrium (LD) between the specified markers by calculating *r* ^2^ value. The single SNP association analyses and haplotype association were also performed by Haploview.

The power for this association study was calculated by using Quanto software version 1.2.4 (http://biostats.usc.edu/software) [32]. The population risk is 0.006 and relative risk was set to 1.5 for power calculation.

## Results

The concordance rate of genotype in the re-genotyped samples by Sequenom was more than 99%. All of these eighteen SNPs in *CACNA1C* were successfully genotyped in 239 nuclear families and polymorphic with minor allele frequency (MAF) more than 5%. None of the genotype distributions of these SNPs in parents and affected children deviated from Hardy-Weinberg equilibrium (S1 Table). The power to detect these risk alleles was ranged from 69% to 86.6% except for rs1006737 and rs4765905 in 239 trios.

Univariate (single marker) test demonstrated that no SNPs were associated with autism in 239 trios. The LD structure constructed from 18 SNPs is shown in S1 Fig. Two SNPs rs1006737 and rs4765905 have a trend of association with autism (*p =* 0.071, 0.096, respectively) (Table 1). The association results calculated by Haploview were similar to those calculated by FBAT (S2 Table). To further confirm the association between rs1006737 and rs4765905 and autism, we expanded the sample size to 553 trios by adding 314 trios. The power to detect risk alleles for rs1006737 and rs4765905 was increased to 58% in 553 trios. None of the genotype distributions of these two SNPs in parents and affected children deviated from Hardy-Weinberg equilibrium in 553 trios (S3 Table).

**Table 1:** Results of family-based association test between 18 SNPs in *CACNA1C* and autism in 239 trios.

Marker	position	Allele	Afreq	Families	S	E (S)	Var (S)	Z	*p*
rs11062065	2011392	C	0.811	126	175.0	175.5	35.25	-0.084	0.933
		T	0.189	126	77.0	76.5	35.25	0.084	
rs917365	2043005	A	0.720	143	181.0	184.0	45.00	-0.447	0.655
		G	0.280	143	105.0	102.0	45.00	0.447	
rs4765663	2069594	C	0.163	111	66.0	63.5	30.75	0.451	0.652
		G	0.837	111	156.0	158.5	30.75	-0.451	
rs1558322	2120889	A	0.253	138	95.0	98.5	42.75	-0.535	0.592
		G	0.747	138	181.0	177.5	42.75	0.535	
rs7298845	2175167	A	0.711	156	203.0	194.0	48.50	1.292	0.196
		G	0.289	156	109.0	118.0	48.50	-1.292	
rs2239031	2227003	G	0.754	141	190.0	182.5	43.75	1.134	0.257
		T	0.246	141	92.0	99.5	43.75	-1.134	
rs1006737	2236129	A	0.066	56	23.0	30.0	15.00	-1.807	0.071
		G	0.934	56	89.0	82.0	15.00	1.807	
rs4765905	2240418	C	0.066	57	24.0	30.5	15.25	-1.664	0.096
		G	0.934	57	90.0	83.5	15.25	1.664	
rs2238060	2316328	A	0.658	158	202.0	194.0	48.50	1.149	0.251
		C	0.342	158	114.0	122.0	48.50	-1.149	
rs2238070	2346949	G	0.540	182	189.0	188.5	63.75	0.063	0.950
		T	0.460	182	175.0	175.5	63.75	-0.063	
rs2238083	2377835	C	0.231	123	82.0	79.0	37.50	0.490	0.624
		T	0.769	123	164.0	167.0	37.50	-0.490	
rs2239062	2393406	G	0.295	156	118.0	118.5	50.25	-0.071	0.944
		T	0.705	156	194.0	193.5	50.25	0.071	
rs2239074	2429383	C	0.796	132	172.0	175.0	39.50	-0.477	0.633
		T	0.204	132	92.0	89.0	39.50	0.477	
rs4765686	2450917	A	0.687	166	198.0	202.0	52.00	-0.555	0.579
		G	0.313	166	134.0	130.0	52.00	0.555	
rs2239109	2519645	G	0.267	152	104.0	104.5	47.25	-0.073	0.942
		T	0.733	152	200.0	199.5	47.25	0.073	
rs2238090	2574166	A	0.295	158	124.0	118.5	49.25	0.784	0.433
		G	0.705	158	192.0	197.5	49.25	-0.784	
rs216008	2611971	C	0.618	166	193.0	192.0	54.00	0.136	0.892
		T	0.382	166	139.0	140.0	54.00	-0.136	
rs6489375	2668602	A	0.339	169	138.0	131.0	52.50	0.966	0.334
		G	0.661	169	200.0	207.0	52.50	-0.966	

Afreq, allele frequency; Families, number of informative families; S, test statistics for the observed number of transmitted alleles;

E(S), expected value of S under the null hypothesis (i.e., no linkage and no association).

Single marker association test demonstrated that G allele of rs1006737 showed a preferential transmission from parents to children affected with autism (G>A, Z = 2.105, *p* = 0.035). Moreover, rs4765905 was nominal significantly associated with autism (G>C, Z = 2.105, *p* = 0.035). Allele frequencies and the results of FBAT for single SNPs analysis are shown in Table 2. The results of each allele transmitted from heterozygous parents to affected children calculated by Haploview are shown in S4 Table.

**Table 2:** Results of association analyses between two SNPs in *CACNA1C* and autism in 553 trios.

Marker	Allele	Afreq	Families	S	E (S)	Var (S)	Z	*p*
rs1006737	A	0.063	124	53.0	65.0	32.5	-2.105	**0.035**
	G	0.937	124	195.0	183.0	32.5	2.105	
rs4765905	C	0.063	124	53.0	65.0	32.5	-2.105	**0.035**
	G	0.937	124	195.0	183.0	32.5	2.105	

Afreq, allele frequency; Families, number of informative families; S, test statistics for the observed number of transmitted alleles;

E(S), expected value of S under the null hypothesis (i.e., no linkage and no association).

We calculated the pairwise LD for all possible pairs of the SNPs. Strong LD block was detected between rs1006737 and rs4765905 (*r* ^2^ = 1). Haplotype analyses showed that haplotype G-G (rs1006737-rs4765905) demonstrated an excess transmission (*p* = 0.030, Global *p* = 0.046). While haplotype constructed from A allele of rs1006737 and C allele of rs4765905 was a protective haplotype (*p* = 0.023, Global *p* = 0.046). To decrease false positive results, we performed permutation test for multiple testing correction. After using permutation test of 10,000 rounds, the results were still significant (*p* = 0.025). The results of specific and global haplotype association are shown in Table 3. Moreover, haplotype association results calculated by Haploview are listed in S5 Table. The genotype data in our study are shown in S6 and S7 Tables.

**Table 3:** Results of association analyses for haplotype constructed from rs1006737 and rs4765905 in *CACNA1C* in 553 trios.

Marker	Haplotypes	freq	Fam	S	E (S)	Var (S)	Z	*p*	Global *p*	Permutation ^a^ *p*
**rs1006737-rs4765905**	**G-G**	0.937	119	190.00	178.00	30.50	2.173	**0.030**	**0.046**	**0.027**
	**A-C**	0.062	118	47.00	59.50	30.25	-2.273	**0.023**		

^a^ Whole marker permutation test using chisq sum *p* value, the number of permutation is 10,000,. freq, Estimation of haplotype frequencies;

Fam, number of informative families; S, test statistics for the observed number of transmitted alleles;

E(S), expected value of S under the null hypothesis (i.e., no linkage and no association).

## Discussion

Previous studies demonstrated that *CACNA1C* was associated with schizophrenia. To test whether *CACNA1C* is involved in the etiology of autism, we performed a family based association study. Our results identified a nominal significant association between two SNPs (rs1006737 and rs4765905) in *CACNA1C* and autism in 553 nuclear families of Chinese Han ancestry. Moreover, haplotype analyses indicated statistically significant association between *CACNA1C* and autism.

However, our study found that G allele of rs1006737 was associated with autism (*p* = 0.035), while the risk allele in schizophrenia was A allele. The inconsistence results might be due to a few reasons. First, one reason was the genetic heterogeneity of ethnicity. The allele frequency of rs1006737 is different between CHB (Han Chinese in Beijing, China) and CEU (Utah residents with Northern and Western European ancestry from the CEPH collection) populations. Our results show that the MAF of rs1006737 is 0.063 in CHB population, while that is about 0.33–0.36 in CEU population [6,9]. The MAF of rs4765905 in CHB is also 0.063. These two SNPs rs4765905 and rs1006737 are in a strong LD block. These results are consistent with those of HapMap project. Second, it might be likely that the genetic signal is tagging a less common (and possibly rare) genetic variant which might contribute directly to autism risk, such as the rare mutation G406R in Timothy Syndrome. Third, the mechanism of genetic etiology of autism and schizophrenia is different despite the susceptibility genes overlap between these two diseases.

Two haplotypes constructed from rs1006737 and rs4765905 which are in a strong LD block were associated with autism. There are quite few other SNPs between these two SNPs. It will be interesting to investigate the association between other SNPs in this region and autism, and to explore whether there are differences of association results between the Chinese population and other population. In future, we will explore the association between autism and SNPs in this region by selecting SNPs in a high intensity. Moreover, it is important to perform mutation screening in *CACNA1C* to detect potentially deleterious rare variants.

Evidence for shared risk was observed for specific genes between schizophrenia and ASD. The susceptibility genes (such as *DISC1* [33,34], *RELN* [35,36], *GABA* [37–39], *SHANK3* [40,41], *NRXN1* [20,42], *NTNG1* [43,44], etc.), which were associated with schizophrenia, also confer risk to ASD. The cross-disorder analyses reveal a significant genetic overlap between schizophrenia and ASD [22,45–49]. Furthermore, epidemiological and neuroimaging studies provided further support for biological overlap between schizophrenia and ASD [50,51]. The most recent evidence for shared etiology comes from studies of rare copy number variants [52]. However, the risk variants were not completely overlap between autism and schizophrenia. These results indicated the existence of shared genetic susceptibility to schizophrenia and autism, suggesting the possibility that the genes may exert their effects through a biological pathway common to both disorders.

Recent studies suggested that calcium channel dysfunction may contribute to the pathogenesis of autism. Three rare missense mutations of *CACNB2* which encodes a subunit of a voltage-dependent calcium channel protein were detected in ASD-affected families. Two of these mutations displayed significantly decelerated time-dependent inactivation as well as increased sensitivity of voltage-dependent inactivation [53]. Another study provided evidence that rs10848653 in *CACNA1C* was associated with ASD [54].

The calcium ion is one of the most versatile and universal of biological signaling molecules [55]. In brain, the subunit encoded by *CACNA1C* is the major constituent of brain L-type voltage gated calcium channels, and is a crucial regulator of dendritic calcium influx in response to synaptic activity [56]. It is most frequently implicated in coupling of cell membrane depolarization to transient increase of the membrane permeability for calcium, leading to activation and potentially changes in intracellular signaling pathway activity, gene transcription, and synaptic plasticity. Therefore, CACNA1C plays important roles in the proper function of numerous neurological circuits including hippocampus, amygdala, and mesolimbic reward system, which are strongly implicated in psychiatric disease pathophysiology [57].

Moreover, neuroimaging researches provided evidence that CACNA1C might affect brain regional activation and inter-regional connectivity. Previous study demonstrated that the effect of rs1006737 in *CACNA1C* on the brain converges on the neural circuitry involved in affect processing [4,58]. Strong evidence indicates that rs1006737 exerts pleiotropic effects on particular brain functions and affects different brain regions (such as amygdala, hippocampus, and ventrolateral prefrontal cortex). Moreover, alteration in *CACNA1C* expression may be a molecular mechanism of genetic risk [12]. There is converging evidence that patients with autism may have affected brain regional activation and inter-regional connectivity [59–64]. A recent study demonstrated that beta connectivity was reduced during emotional face processing in adolescents with autism [65]. These findings suggest that functional disconnection in brain networks mediating emotional processes may contribute to deficits in social cognition in ASD. CACNA1C might potentially be related to alternations in intracellular calcium homeostasis and then confer risk of autism.

Other replication studies are needed. In addition, further studies are necessary to understand the underlying mechanisms the gene *CACNA1C* exerts on autism as well as other psychiatric disorders.

## Conclusions

Our study indicates that *CACNA1C* is associated with autism in Han Chinese population. *CACNA1C* might play a role in the pathogenesis of autism.

## Supporting Information

S1 Fig Linkage disequilibrium block constructed from 18 SNPs in *CACNA1C*. Markers with linkage disequilibrium (0<*r* ^2^≤1) are shown in black through grey (color intensity decreases with decreasing *r* ^2^ value). The square is shown in black when *r* ^2^ = 1, while the square is white when *r* ^2^ = 0.
(DOC)

S1 Table Information of 18 SNPs in *CACNA1C* and genotype frequencies in 239 autism trios. ^a^ Hardy-Weinberg equilibrium *p* value for genotype distributions in children affected with autism; ^b^ Hardy-Weinberg equilibrium *p* value for genotype distributions in parents.
(DOC)

S2 Table Association results of 18 SNPs in *CACNA1C* and autism in 239 trios calculated by Haploview. SNPs, single nucleotide polymorphisms; Overtransmitted, the allele overtransmitted to affected offspring; T, transmitted; U, untransmitted; T:U is the ratio of transmissions to non transmissions of the overtransmitted allele.
(DOC)

S3 Table Genotype frequencies of rs1006737 and rs4765905 in 553 autism trios. ^a^ Hardy-Weinberg equilibrium *p* value for genotype distributions in children affected with autism; ^b^ Hardy-Weinberg equilibrium *p* value for genotype distributions in parents.
(DOC)

S4 Table Association analyses of two SNPs (rs1006737 and rs4765905) in 553 trios calculated by Haploview. SNPs, single nucleotide polymorphisms; Overtransmitted is the allele overtransmitted to affected offspring; T, transmitted; U, untransmitted; T:U is the ratio of transmissions to non transmissions of the overtransmitted allele.
(DOC)

S5 Table Haplotype analyses of two haplotypes constructed from rs1006737 and rs4765905 in 553 trios calculated by Haploview. ^a^ the number of permutation is 10,000; SNPs, single nucleotide polymorphisms; Freq, frequency; T, transmitted; U, untransmitted; T:U is the ratio of transmissions to non transmissions of the overtransmitted allele.
(DOC)

S6 Table Genotyping data of the selected 18 SNPs in *CACNA1C* in 239 trios of Han Chinese descent. (XLS)

S7 Table Genotyping data of rs1006737 and rs4765905 in additional 314 trios of Han Chinese descent. (XLS)
