# Supplementary material for: Schizophrenia Related Variants in CACNA1C also Confer Risk of Autism
Source: PLoS One. 2015 Jul 23;10(7):e0133247. doi: 10.1371/journal.pone.0133247 (PMC4512676; doi:10.1371/journal.pone.0133247)
Supplement: S1 Fig — Markers with linkage disequilibrium (0<r 2≤1) are shown in black through grey (color intensity decreases with decreasing r 2 value). The square is shown in black when r 2 = 1, while the square is white when r 2 = 0. (DOC) [file pone.0133247.s001.doc]

**S1 Fig. Linkage disequilibrium block constructed from 18 SNPs in *CACNA1C***


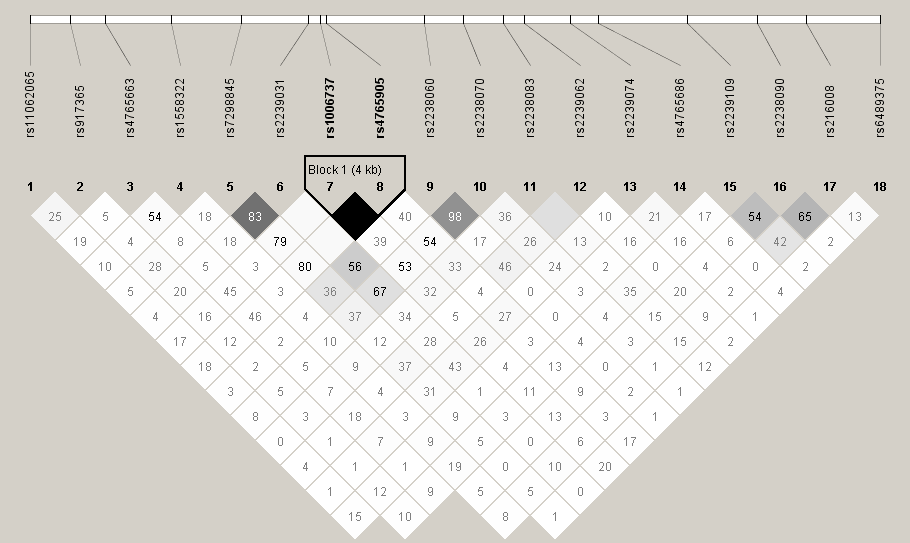


Markers with linkage disequilibrium (0<*r*^2^≤1) are shown in black through grey (color intensity decreases with decreasing *r*^2^ value).

The square is shown in black when *r*^2^=1, while the square is white when *r*^2^=0.
