# Supplementary material for: Schizophrenia Related Variants in CACNA1C also Confer Risk of Autism
Source: PLoS One. 2015 Jul 23;10(7):e0133247. doi: 10.1371/journal.pone.0133247 (PMC4512676; doi:10.1371/journal.pone.0133247)
Supplement: S1 Table — a Hardy-Weinberg equilibrium p value for genotype distributions in children affected with autism; b Hardy-Weinberg equilibrium p value for genotype distributions in parents. (DOC) [file pone.0133247.s002.doc]

**S1 Table.** **Information of 18 SNPs in *CACNA1C* and genotype frequencies in 239 autism trios**

| **Marker** | **Genotype frequencies in children** | | | ***p* _HWE_ ^a^** | **Genotype frequencies in parents** | | | ***p* _HWE_ ^b^** |
| --- | --- | --- | --- | --- | --- | --- | --- | --- |
| rs11062065 | CC | CT | TT | 0.78 | CC | CT | TT | 0.39 |
|  | 156 | 75 | 8 |  | 317 | 141 | 20 |  |
| rs917365 | AA | AG | GG | 0.46 | AA | AG | GG | 0.21 |
|  | 120 | 102 | 17 |  | 252 | 181 | 43 |  |
| rs4765663 | CC | CG | GG | 0.12 | CC | CG | GG | 0.62 |
|  | 3 | 71 | 162 |  | 11 | 131 | 328 |  |
| rs1558322 | AA | AG | GG | 0.27 | AA | AG | GG | 0.29 |
|  | 18 | 83 | 138 |  | 35 | 172 | 271 |  |
| rs7298845 | AA | AG | GG | 0.39 | AA | AG | GG | 0.80 |
|  | 130 | 89 | 20 |  | 243 | 194 | 41 |  |
| rs2239031 | GG | GT | TT | 0.22 | GG | GT | TT | 0.78 |
|  | 145 | 78 | 16 |  | 273 | 175 | 30 |  |
| rs1006737 | AA | AG | GG | 0.39 | AA | AG | GG | 0.44 |
|  | 0 | 25 | 214 |  | 1 | 60 | 416 |  |
| rs4765905 | CC | CG | GG | 0.39 | CC | CG | GG | 0.42 |
|  | 0 | 25 | 214 |  | 1 | 61 | 416 |  |
| rs2238060 | AA | AC | CC | 0.20 | AA | AC | CC | 0.25 |
|  | 111 | 96 | 30 |  | 213 | 201 | 60 |  |
| rs2238070 | GG | GT | TT | 0.76 | GG | GT | TT | 0.10 |
|  | 69 | 121 | 49 |  | 130 | 255 | 92 |  |
| rs2238083 | CC | CT | TT | 0.21 | CC | CT | TT | 0.09 |
|  | 11 | 94 | 126 |  | 16 | 169 | 270 |  |
| rs2239062 | GG | GT | TT | 0.43 | GG | GT | TT | 0.80 |
|  | 18 | 104 | 117 |  | 41 | 202 | 235 |  |
| rs2239074 | CC | CT | TT | 0.20 | CC | CT | TT | 0.63 |
|  | 152 | 73 | 14 |  | 301 | 158 | 18 |  |
| rs4765686 | AA | AG | GG | 0.53 | AA | AG | GG | 0.71 |
|  | 107 | 109 | 23 |  | 224 | 209 | 45 |  |
| rs2239109 | GG | GT | TT | 0.48 | GG | GT | TT | 0.81 |
|  | 19 | 89 | 131 |  | 33 | 189 | 256 |  |
| rs2238090 | AA | AG | GG | 0.85 | AA | AG | GG | 0.89 |
|  | 22 | 103 | 114 |  | 42 | 197 | 238 |  |
| rs216008 | CC | CT | TT | 0.53 | CC | CT | TT | 0.38 |
|  | 90 | 117 | 32 |  | 186 | 217 | 75 |  |
| rs6489375 | AA | AG | GG | 0.65 | AA | AG | GG | 0.84 |
|  | 31 | 105 | 101 |  | 56 | 212 | 209 |  |

^a^ Hardy-Weinberg equilibrium *p* value for genotype distributions in children affected with autism;

^b^ Hardy-Weinberg equilibrium *p* value for genotype distributions in parents.
