# Supplementary material for: Schizophrenia Related Variants in CACNA1C also Confer Risk of Autism
Source: PLoS One. 2015 Jul 23;10(7):e0133247. doi: 10.1371/journal.pone.0133247 (PMC4512676; doi:10.1371/journal.pone.0133247)
Supplement: S2 Table — SNPs, single nucleotide polymorphisms; Overtransmitted, the allele overtransmitted to affected offspring; T, transmitted; U, untransmitted; T:U is the ratio of transmissions to non transmissions of the overtransmitted allele. (DOC) [file pone.0133247.s003.doc]

**S2 Table. Association results of 18 SNPs in *CACNA1C* and autism in 239 trios calculated by Haploview**

| **SNPs** | **Overtransmitted** | **T : U** | **Chi Square** | ***p*** |
| --- | --- | --- | --- | --- |
| rs11062065 | T | 71 : 70 | 0.007 | 0.933 |
| rs917365 | G | 93 : 87 | 0.200 | 0.655 |
| rs4765663 | C | 64 : 59 | 0.203 | 0.652 |
| rs1558322 | G | 89 : 82 | 0.287 | 0.592 |
| rs7298845 | A | 106 : 88 | 1.670 | 0.196 |
| rs2239031 | G | 95 : 80 | 1.286 | 0.257 |
| rs1006737 | G | 37 : 23 | 3.267 | 0.071 |
| rs4765905 | G | 37 : 24 | 2.770 | 0.096 |
| rs2238060 | A | 105 : 89 | 1.320 | 0.251 |
| rs2238070 | G | 128 : 127 | 0.004 | 0.950 |
| rs2238083 | C | 78 : 72 | 0.240 | 0.624 |
| rs2239062 | T | 101 : 100 | 0.005 | 0.944 |
| rs2239074 | T | 82 : 76 | 0.228 | 0.633 |
| rs4765686 | G | 108 : 100 | 0.308 | 0.579 |
| rs2239109 | T | 95 : 94 | 0.005 | 0.942 |
| rs2238090 | A | 104 : 93 | 0.614 | 0.433 |
| rs216008 | C | 109 : 107 | 0.019 | 0.892 |
| rs6489375 | A | 112 : 98 | 0.933 | 0.334 |

SNPs, single nucleotide polymorphisms; Overtransmitted is the allele overtransmitted to affected offspring; T, transmitted; U, untransmitted; T:U is the ratio of transmissions to non transmissions of the overtransmitted allele.
