# Supplementary material for: Schizophrenia Related Variants in CACNA1C also Confer Risk of Autism
Source: PLoS One. 2015 Jul 23;10(7):e0133247. doi: 10.1371/journal.pone.0133247 (PMC4512676; doi:10.1371/journal.pone.0133247)
Supplement: S3 Table — a Hardy-Weinberg equilibrium p value for genotype distributions in children affected with autism; b Hardy-Weinberg equilibrium p value for genotype distributions in parents. (DOC) [file pone.0133247.s004.doc]

**S3 Table.** **Genotype frequencies of rs1006737 and rs4765905 in 553 autism trios**

| **Marker** | **Chr. position** | **Genotype frequencies in children** | | | ***p* _HWE_ ^a^** | **Genotype frequencies in parents** | | | ***p* _HWE_ ^b^** |
| --- | --- | --- | --- | --- | --- | --- | --- | --- | --- |
| rs1006737 | 2215556 | AA | AG | GG | 0.19 | AA | AG | GG | 0.24 |
|  |  | 0 | 58 | 486 |  | 2 | 132 | 953 |  |
| rs4765905 | 2219845 | CC | CG | GG | 0.19 | CC | CG | GG | 0.24 |
|  |  | 0 | 57 | 487 |  | 2 | 132 | 953 |  |

^a^ Hardy-Weinberg equilibrium *p* value for genotype distributions in children affected with autism;

^b^ Hardy-Weinberg equilibrium *p* value for genotype distributions in parents.
