# Supplementary material for: Schizophrenia Related Variants in CACNA1C also Confer Risk of Autism
Source: PLoS One. 2015 Jul 23;10(7):e0133247. doi: 10.1371/journal.pone.0133247 (PMC4512676; doi:10.1371/journal.pone.0133247)
Supplement: S4 Table — SNPs, single nucleotide polymorphisms; Overtransmitted is the allele overtransmitted to affected offspring; T, transmitted; U, untransmitted; T:U is the ratio of transmissions to non transmissions of the overtransmitted allele. (DOC) [file pone.0133247.s005.doc]

**S4 Table. Association analyses of two SNPs (rs1006737 and rs4765905) in 553 trios calculated by Haploview**

| **SNPs** | **Overtransmitted** | **T : U** | **Chi Square** | ***p*** |
| --- | --- | --- | --- | --- |
| rs1006737 | G | 77 : 53 | 4.431 | 0.035 |
| rs4765905 | G | 77 : 53 | 4.431 | 0.035 |

SNPs, single nucleotide polymorphisms; Overtransmitted is the allele overtransmitted to affected offspring; T, transmitted; U, untransmitted; T:U is the ratio of transmissions to non transmissions of the overtransmitted allele.
