# Supplementary material for: Schizophrenia Related Variants in CACNA1C also Confer Risk of Autism
Source: PLoS One. 2015 Jul 23;10(7):e0133247. doi: 10.1371/journal.pone.0133247 (PMC4512676; doi:10.1371/journal.pone.0133247)
Supplement: S5 Table — a the number of permutation is 10,000; SNPs, single nucleotide polymorphisms; Freq, frequency; T, transmitted; U, untransmitted; T:U is the ratio of transmissions to non transmissions of the overtransmitted allele. (DOC) [file pone.0133247.s006.doc]

**S5 Table. Haplotype analyses of two haplotypes constructed from rs1006737 and rs4765905**

**in 553 trios calculated by Haploview**

| **Haplotype** | **Freq** | **T : U** | **Chi Square** | ***p*** | **Permutation^a^ *p*** |
| --- | --- | --- | --- | --- | --- |
| rs1006737- rs4765905 |  |  |  |  |  |
| G-G | 0.937 | 77.0 : 53.0 | 4.431 | 0.035 | 0.038 |
| A-C | 0.063 | 53.0 : 77.0 | 4.431 | 0.035 | 0.038 |

^a^ the number of permutation is 10,000; SNPs, single nucleotide polymorphisms; Freq, frequency;

T, transmitted; U, untransmitted; T:U is the ratio of transmissions to non transmissions of the overtransmitted allele.
